# Ethogram of the Chinese Giant Salamander during the Breeding Period Based on the PAE Coding System

**DOI:** 10.3390/ani13233632

**Published:** 2023-11-23

**Authors:** Shouliang Luo, Pei Wang, Yifang Zhang, Ziteng Wang, He Tian, Qinghua Luo

**Affiliations:** 1 Hunan Engineering Laboratory for Chinese Giant Salamander’s Resource Protection and Comprehensive Utilization, School of Biological Resources and Environmental Sciences, Jishou University, Zhangjiajie 427000, China; luosl2021@163.com (S.L.); wangpei0229@126.com (P.W.); zyf33629@163.com (Y.Z.); 16681982610@163.com (Z.W.); tianhe970808@126.com (H.T.); 2 College of Biological and Chemical Engineering, Changsha University, Changsha 410022, China

**Keywords:** Chinese giant salamander (*Andrias davidianus*), breeding period, ethogram, PAE coding, action, posture

## Abstract

**Simple Summary:**

Constructing an ethogram is the basis and prerequisite for an in-depth study of animal behavior and its complex relationship with the environment. The Chinese giant salamander (*Andrias davidianus*) is the flagship species of endangered amphibians. In order to understand all of the behaviors and ecological laws of *A. davidianus* during the breeding period, this study monitored the behavior and related environmental factors of *A. davidianus* during its breeding period and constructed an ethogram of *A. davidianus* during the breeding period based on the PAE (Posture-Act-Environment) coding system. To accurately indicate the environmental conditions and thresholds required for reproductive behavior, quantitative data on water quality and habitat factors that have a significant impact on *A. davidianus*’s behavior were included in the coding framework, such as water temperature (WT), pH, dissolved oxygen (DO), etc. The ethogram included all behaviors of *A. davidianus* during the breeding period for the first time, totaling 45 behavioral patterns, grouped into 9 categories, which were discriminated and coded based on the corresponding posture, action, and environmental factors when each behavioral pattern occurred. The results lay a foundation for revealing the ecological law of *A. davidianus*’s reproductive behavior and the suitable habitat; provide a scientific basis for optimizing the breeding technology of *A. davidianus* and protecting its wild population; and provide a reference for quantitative ethogram research on amphibians.

**Abstract:**

The PAE (Posture-Act-Environment) coding system is a behavior coding system that divides the study of animal behavior into postures, actions, and the corresponding environmental factors, and they are coded correspondingly. It determines the analysis dimension to standardize the study of behavior. To investigate the behavior of *A. davidianus* during the breeding period, as well as their related postures, actions, and required environmental conditions, this study monitored the behavior of four pairs of *A. davidianus* in a simulated natural breeding pool using an infrared image monitoring system and recorded the changes in water quality during this process using a water quality monitoring system. The process of reproductive behaviors was observed and recorded with the random sampling method and the focal animal sampling method to classify and code the behaviors, and the ethogram of *A. davidianus* during the breeding period was constructed based on the PAE coding system. The result showed that 10 postures, 33 actions, 11 environments, and 45 behavioral patterns were differentiated and defined, which were classified into 9 categories of behaviors according to the behavioral function. Among these categories, five were distinguished as behaviors unique to the reproductive period, which include sand pushing, showering, courtship, oviposition, and parental care. The remaining four categories were daily behaviors: exercise, feeding, rest, and miscellaneous behaviors. The quantitative data on water quality and habitat factors that had a significant impact on the behavior of *A. davidianus*, such as water temperature (WT), pH, and dissolved oxygen (DO), were included in the coding framework, which more accurately expresses the environmental conditions and thresholds required for the breeding behavior.

## 1. Introduction

The ethogram is the catalog, which is compiled based on the identification and classification of animal behavior. It is a fundamental tool for studying animal behavior. Compiling an ethogram involves the induction of all animal behaviors and mastering the characteristics of animal behavior. It is an important basic part of behavioral research [1,2] and the starting point for all behavioral studies [3]. It is helpful to understand the internal relationship between animal behavior and its functions, as it promotes an understanding of the ecological functions of animal behavior [4] and also provides guidance for animal protection [5].

A traditional ethogram involves the classification, nomenclature, and descriptive definition of various animal behaviors. Among the tailed amphibian species, this kind of ethogram has been built, such as the aggressive, sexual, social, and locomotory behaviors of Hellbender (*Cryptobranchus alleganiensis*) [6], the paternal care behavior of Japanese giant salamanders (*Andrias japonicus*) [7], the courtship behavior of Taliang crocodile newt (*Liangshantriton taliangensis*) [8], Panha’s crocodile newt (*Tylototriton panhai*) [9], etc. Jiang proposed the PAE (Posture-Act-Environment) coding system, which divides the study of animal behavior into postures, actions, and their required environmental elements and encodes them in order to standardize behavioral data. This has formalized the dimensions of behavioral studies and hierarchical behavioral analyses. It is convenient to store behavior data in data recorders and computers for analysis and processing [4]. The system has been widely used in beasts, such as the Amur tiger (*Panthera tigris altaica*) [10] and the mainland serow (*Capricornis sumatraensis*) [11]; birds, such as the painted snipe (*Rostratula benghalensis*) [12] and scaly-sided merganser (*Mergus squamatus*) [13]; and reptiles, such as the toad-headed lizard (*Phrynocephalus vlangalii*) [14]. It has also been reported in aquatic animals, such as Yangtze finless porpoises (*Neophocaena phocaenoides asiaeorientalis*) [15] and *Schizothorax wangchiachii* [16]. However, few studies have been reported on the ethogram of amphibians in the PAE coding system.

The Chinese giant salamander (*Andrias davidianus*), the largest extant amphibian species in the world, is a cryptobranchid salamander endemic to China. Studies have shown that the Chinese giant salamander comprises four species: *Andrias davidianus* [17], *Andrias sligoi* [18], *Andrias jiangxiensis* sp. nov. [19], and *Andrias cheni* sp. nov. [20]. However, most experts believe that these are scientific explorations, and the confirmation of these species requires more studies involving reproductive isolation. It has been widely distributed in the Yangtze, Yellow River, and Pearl River basins in China [21], including 18 provinces or equivalent administrative regions [22]. However, since the 1950s, the wild population of *A. davidianus* has significantly declined [23]. There are three main reasons: the destruction and loss of habitats, such as building dams and cutting down forests, making habitats fragmented and landed; trafficking in wild populations, which leads to overharvesting; and environmental pollution caused by excessive use of pesticides and fertilizers [21,24]. It was designated as a Grade II protected wild animal in China in 1998 and has been listed as Critically Endangered by the International Union for Conservation of Nature (IUCN) [25]. Currently, breeding *A. davidianus* has formed an industry in China, so the National Forestry and Grassland Administration of China adjusted the list of protected animals in 2021, and only *A. davidianus* in the wild is listed as a Grade II protected animal [26]. In China, in order to rescue *A. davidianus*, 53 protected areas involving this species have been established [24], and the species has been effectively restored through artificial proliferation and release.

Most existing research on *A. davidianus*’s behavior only qualitatively describes its behavior categories. Zhang et al. found four oviposition behaviors of *A. davidianus*: kissing, knocking bellies, playing, and caudal mating [27]. Liang et al. preliminary observed and described five categories of *A. davidianus* reproductive behaviors: sand pushing, courtship, showering, parental care, and mating [28]. A few quantitative studies on *A. davidianus*’s behavior have been conducted. Luo et al. systematically reported five categories of reproductive behaviors of *A. davidianus*, including sand pushing, showering, courtship, oviposition, and parental care, and quantified the frequency [29]. In addition, the relationship between spawning, parental care, and water quality was also analyzed [30]. Xu established a pre-breeding behavior coding system (PAE), but it did not include spawning and parental care, which are the key reproductive behaviors of *A. davidianus* [31]. How many behaviors of *A. davidianus* occur in the breeding period? What environmental conditions can lead to these behaviors? What are the behaviorally important roles in successful reproduction? This study used an infrared video monitoring system and a water quality monitoring system to obtain video information and water quality data on the behavior of *A. davidianus* throughout the breeding season. Based on these data, we constructed an ethogram and a PAE coding system for behavior during the breeding period of *A. davidianus*. The research results will help us to understand the behavior pattern of *A. davidianus* in the breeding period and their relationship with the ecological environment, provide a scientific basis for optimizing the breeding technology and protecting the wild population of *A. davidianus*, and provide a reference for quantitative behavior research in amphibians.

## 2. Materials and Methods

### 2.1. Study Site and Observation Subjects

**Figure 1:**
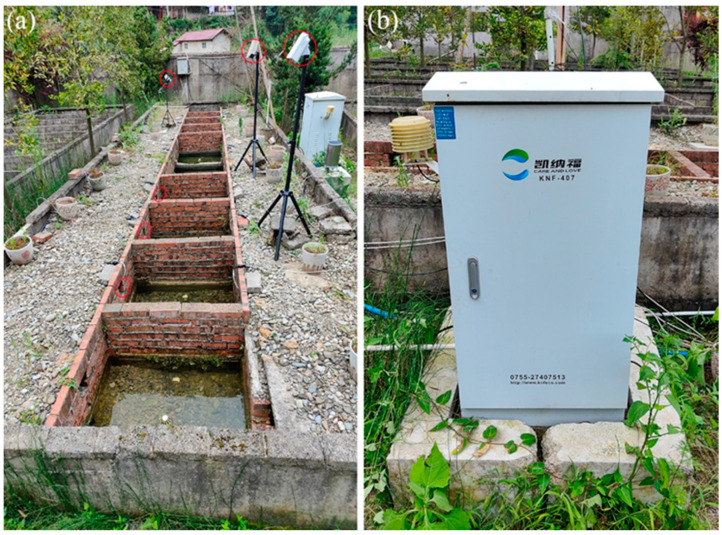
Simulated natural breeding pool (**a**) and water quality monitoring system (**b**). These red circles are camera locations.

In China, the breeding of *A. davidianus* has been industrialized. This effectively reduces the illegal capture of this wild population and provides resources for the artificial proliferation and release of this species. At present, the breeding methods of *A. davidianus* in companies are divided into artificial and simulated natural reproduction. The research site was located in the ecological breeding base of Zhangjiajie Zhuyuan Giant Salamander Biotechnology Co., Ltd. (29°25′56″ N, 110°22′55″ E, altitude: 471 m) in Tangxiyu Village, Kongkeshu Township, Sangzhi County, Hunan Province. Simulated natural breeding pools comprised an artificial stream with caves on both sides (Figure 1a).

The breeding period of *A. davidianus* refers to the time since sexually mature *A. davidianus* exhibits unique reproductive behaviors, such as sand pushing, showering, courtship, oviposition, and parental care, which are mainly concentrated in the summer and autumn. Taking the oviposition time as the boundary, the breeding period of *A. davidianus* was divided into prophase and anaphase [31]. The breeding period of *A. davidianus* in this ecological breeding base occurs from July to October every year.

Eight *A. davidianus* individuals (4♂, 4♀) were selected, aged 7–11 years, with a body length ranging from 1.03 to 1.28 m and a body weight ranging from 6.9 to 12.6 kg.

### 2.2. Data Acquisition

Infrared cameras (Hikvision DS-2cd3T5d-i5, Hangzhou Haiji Visual Digital Technology Co., Ltd., Hangzhou, China) were installed on the cave side and above the stream to monitor and collect video data on *A. davidianus*’s behavior (Figure 1a). Additionally, a water quality monitoring system (KFN-407, Shenzhen Kainafu Technology Co., Ltd., Shenzhen, China) (Figure 1b) was utilized to monitor the changes in water quality indicators in streams (measured every 30 min), including water temperature (WT), dissolved oxygen (DO), pH, etc. The entire experimental monitoring took place from July to October in 2020–2022.

### 2.3. Behavior Coding

For the collected behavioral video, we observed 1 d at 2 d intervals and collected information on each behavioral pattern, including the occurrence time periods, age-sex group, frequency, etc. According to behavioral function, these behavioral patterns were classified. Referring to the behavioral PAE coding methods of Chen et al. [15], Zhu et al. [16], and Luo et al. [32], the hierarchy of *A. davidianus*’s behavior was analyzed in terms of postures, actions, and environments. “Posture” is the state and position of the major body structural parts that *A. davidianus* maintains for a certain period. “Action” refers to the movement of part of the skeletal muscles of an *A. davidianus*’s body over a short period, which causes movement, contraction, diastole, flexion, and displacement of part of the body’s structure. “Environment” indicates the specific surroundings in which each activity of *A. davidianus* occurs, which can be categorized into two main types: biotic environment and abiotic environment. Data on water quality (WT, DO, and pH) throughout the experimental phase were analyzed to determine the range of variation of each data point. Finally, combining the biological and abiotic environmental data in the simulated ecological stream area to construct an environmental code comprehensively.

Based on the random sampling method and the focal animal sampling method, we randomly select a part (about 20%) from the marked video of each behavioral pattern to focus on observation and hierarchically analyze its corresponding postures, actions, and environments. Finally, we integrated the month, frequency, and age-sex groups of each behavioral pattern to establish the ethogram of *A. davidianus* during the breeding period based on the PAE coding system.

## 3. Results

### 3.1. Posture Coding

Ten postures of *A. davidianus* were distinguished and recorded, including lying, groveling, bracing, head-exposing, bending, flipping, crawling, digging, swimming, and leaping (Table 1). These postures were classified into two categories: static postures, which include lying, groveling, bracing, head-exposing, and bending, and dynamic postures, which consist of flipping, crawling, digging, swimming, and leaping.

### 3.2. Action Coding

Thirty-three actions were identified, documented, and coded according to *A. davidianus*’s body position, including the mouth, head, trunk, tail, and limbs (Table 2).

### 3.3. Environmental Coding

A total of 11 environmental factors were identified and classified into biotic and abiotic categories, with the specific attributes of each environment quantified for analysis (Table 3).

### 3.4. Reproductive Ethogram and PAE Coding System

**Figure 2:**
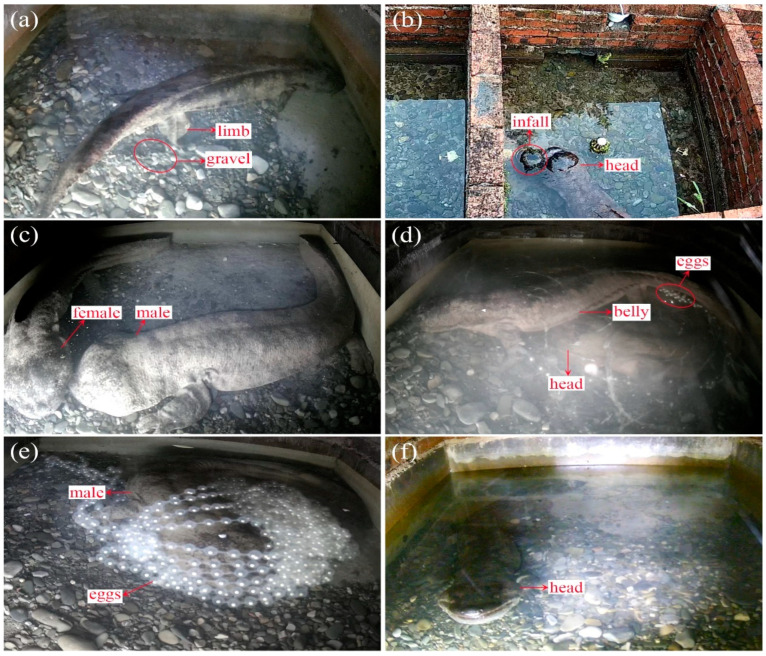
Partial reproductive behavior of *A. davidianus*: (**a**) The male uses his limbs to push sand and pebbles out of the cave; (**b**) The male showers his head at the water inlet; (**c**) The male kisses the female; (**d**) The male uses his head to push against the female’s belly; (**e**) The male is stirring the eggs; (**f**) Breathing with head above water.

This study documented 45 behavioral patterns during the breeding period of *A. davidianus*, which were grouped into 9 categories based on behavioral function. Among them, five categories, including sand pushing, showering, courtship, oviposition, and parental care, are unique to the breeding period of *A. davidianus*. The remaining four categories are daily behaviors, including locomotion, ingestion, resting, and miscellaneous behaviors. The various categories of behaviors are defined as follows:

(1) Sand pushing: The male pushed gravel outward from the cave’s bottom with his head (Figure 2a), limbs, body, and tail.
(2) Showering: The male crawled towards the water inlet, and different parts of their body, including the head, trunk, tail, bending, and head lifting, were continuously showered by the flowing water (Figure 2b).
(3) Courtship: The male displayed an array of behaviors aimed at attracting and stimulating the female, such as knocking bellies side-by-side, riding, kissing (Figure 2c), following, etc.
(4) Oviposition: The female and the male engage in a series of activities such as riding, kissing, knocking bellies (Figure 2d), intertwining, cloacal sniffing (Video S1), and fondling; the male ejaculates, and the female ovulates during the process.
(5) Parental Care: The female leaves the cave after spawning, and the male cares for its offspring by tail fanning, agitating (Figure 2e) (Video S2), shaking, and egg-eating.
(6) Locomotion: The daily displacement behavior of *A. davidianus*, such as chasing, swimming, retreating, etc.
(7) Ingestion: *A. davidianus*’s daily foraging behavior includes catching and swallowing.
(8) Resting: *A. davidianus* maintains the same posture with its whole body in a relaxed state, such as bent resting, head-exposed resting, and lying resting.
(9) Miscellaneous: At various stages, *A. davidianus* may exhibit behaviors such as vigilance, turning over, and breathing (Figure 2f).

The postural codes, action codes, and environmental codes corresponding to the 45 behavioral patterns in 9 categories during the reproduction period of *A. davidianus*, as well as the month, frequency, and age-sex group in which each type of behavior occurs, were matched and coded, and finally synthesized to obtain the ethogram of *A. davidianus* during the reproductive period based on the PAE coding system (Table A1). The results showed that the entire breeding period of the giant salamander is from July to October each year, and male adult activities mostly dominate various behaviors, and most of them mainly occur in caves.

## 4. Discussion

### 4.1. Reproductive Behavior of A. davidianus

Reproduction is a crucial physiological process by which animals continue their species by producing offspring. Animals adjust their reproduction behavior according to environmental and physiological conditions, thereby forming behavioral patterns with specific conditions [33]. This study recorded the unique behaviors of *A. davidianus* during the breeding period, including sand pushing, showering, courtship, oviposition, and parental care.

Sand-pushing behavior may be necessary for the pre-spawning stage. The male modifies the cave’s bottom, creating a larger space for spawning, and increases the water depth inside the cave to create an excellent environment for fertilization and incubation [28,29]. This behavior may eliminate organic matter in the cave and improve the water quality to attract females; the depression in the cave also reduces the risk of the eggs being washed away by flowing water [34]. The showering behavior is predominantly observed in males, which probably promotes the development of the testis and is beneficial to the natural reproduction of adult *A. davidianus* [35]. There is a difference in the growth of males and females, with males growing slightly faster than females of *A. davidianus* [36]. Through courtship, the male transmits pheromones produced by the skin glands to the female to promote the development of the female gonads, and the male also attracts and stimulates the female to mate with intermittent physical contact, resulting in sexual behavior and achieving reproductive synchronization [29]. Showering and courtship have a strong positive correlation [35]. The male’s behavior (e.g., knocking bellies, intertwining, fondling) encourages females to release eggs throughout the oviposition process. After oviposition, the male takes care of the egg and offspring by tail-fanning, agitating, shaking, and egg-eating. Tail fanning, agitation, and shaking enhance oxygen into the water from the air, thereby increasing the oxygen levels within the egg stacks, which satisfy the necessary oxygen supply for the development of embryos. The egg-eating behavior promptly eliminates unfertilized, yolk-stuck, and water-mold-infected eggs, which minimizes the risk of contaminating adjacent eggs [29,37].

### 4.2. PAE Coding System and Ethogram

This study constructed an ethogram of *A. davidianus* based on the PAE coding system during the breeding period. The PAE coding of behavior aims to standardize the dimensions of behavior analysis and facilitate our understanding of the relationship between behavior and environment. However, current studies on the ethogram of cryptobranchiidae species are mostly qualitative [6,7,37,38] and tend to include descriptive linguistic introductions of behaviors. These are easy to study, but descriptions of the same behavior may vary among scholars [10] and lack the specific details and analytical dimensions of behavioral categorization. The PAE coding system divides and codes animal behavior into postures, actions, and corresponding environmental elements. For example, the head-pushing sand behavior of *A. davidianus* mainly occurs in the early breeding period from July to August, under environmental conditions in which the sand and pebbles in the cave are not cleared (environmental code: 1, 4, 5, 9). Sometimes, males crawl (posture code: 7) with their heads lowered, swung, and extended forward while their limbs are bent and swung back and forth (action code: 2, 3, 4, 15, 17, 18). This animal behavior research model benefits the standardization of animal behavioral studies, resource sharing, and comparative analysis of behavioral evolutionary adaptations among species [39]. It also allows for a more definitive analysis of the physiological and ecological benefits of the animals and the effects of environmental conditions [40].

The ethogram identified and defined 9 categories and 45 behaviors of *A. davidianus*. Compared with the ethogram of *A. davidianus* constructed by Xu [31], five categories of behaviors were added, including sand pushing, oviposition, parental care, ingestive, and miscellaneous behaviors. The number of behavioral patterns of *A. davidianus* is similar to that of aquatic animals (43 patterns of *Neophocaena phocaenoides asiaeorientalis* [15] and 46 patterns of *Schizothorax wangchiachii* [16]). However, when compared with reptiles (66 patterns of *Sacalia bealei* [32] and 83 patterns of *Phrynocephalus vlangalii* [14]), mammals (98 patterns of *Panthera tigris altaica* [10]; 78 patterns of *Capricornis sumatraensis* [11]), and birds (83 patterns of *Rostratula benghalensis* [12]; 120 patterns of *Mergus squamatus* [13]), there is a greater difference in the number of behavioral patterns among them. This difference may be related to their evolutionary rank and living environment [16]. *A. davidianus* belongs to the Cryptobranchidae family, and its body structure has not changed obviously since 160 million years ago by fossil studies, so it is called an “aquatic living fossil” [41]. It mainly lives underwater, and its environment is relatively simple. It completes various behavioral activities mainly with its head, limbs, and tail. In quantitative behavior analysis, the same behavior may have different behavioral functions when it occurs at different times and in different subjects [12]. For instance, the behavior of “knocking bellies” during courtship is used by males to attract females’ attention and promote their gonadal development. But it stimulates the female to ovulate when it occurs in the spawning stage. The “vigilance” behavior primarily functions for males to shield females from external threats outside the burrow during the spawning process. However, in other instances, either females or males guard the cave with this behavior, which occurs daily. In summary, the ecological function of behaviors could be effectively identified by comparing the PAE coding components of each behavior.

### 4.3. The Ecological Law of Reproductive Behavior

When constructing the PAE coding system for *A. davidianus*’s behavior, we quantified various environmental factors for the first time. We incorporated them into the environmental coding framework (Table 3), which more accurately expresses the environmental conditions and thresholds required during the reproductive period and is an exploration of a quantitative research model of animal behavior. The family of Cryptobranchidae can successfully breed in a simulated natural environment [6,29,34], and a suitable space can improve the reproductive success rate of *C. alleganiensis*. The confined and narrow environment inhibits the development of the gonads of *A. davidianus*, resulting in a decline in the quality of sperm and eggs [42]. Our study found that *A. davidianus* could perform all necessary activities during the breeding season within caves ranging in size from 0.9 to 1.5 m^2^, with cave entrances measuring 0.25 to 0.35 m in width and 0.3 to 0.35 m in height. This cave size is suitable for *A. davidianus* to freely enter and exit, and it is also beneficial for defending against enemies. Moreover, it reduces the amount of incoming light, which meets the needs of *A. davidianus*, as this species requires a suitable dark environment. The sand and pebbles are covered above the caves, which can reduce the turbidity of the water from rainwater washout, meeting *A. davidianus*’s need for clean water [43]. Water conditions are critical for amphibian development. *A. davidianus* primarily inhabits water, and larval development is also accomplished in water. During the breeding period, the water depth should be maintained at 0.3 to 0.4 m, which provides a suitable water environment for the breeding activities of *A. davidianus*.

*A. davidianus* naturally reproduces from July to September every year, with the reproduction peak typically occurring in August [44], and the incubation period extends from August to mid-October [45]. Our study found that the spawning period of *A. davidianus* occurs mainly in August, which is consistent with the above report. *A. japonicus* spawns from late July to early September [46]. The spawning season of *C. alleganiensis* occurs mainly in September and October but also extends into December and January of the following year [47]. For poikilothermic animals, when the ambient temperature is higher than the starting temperature of their gonadal development, the gonads begin to develop, which is the effective temperature, and the maturation of the gonads depends on the accumulation of the effective temperature. In summary, the breeding season of cryptobranchid salamanders is relatively concentrated, mainly in late summer and autumn. During this period, the temperatures are suitable for the occurrence of reproductive behavior in cryptobranchid salamanders, and they also reach the effective cumulative temperature for the development of their gonads.

Environmental temperature affects the behavioral characteristics of animals. A temperature that is either too high or too low can affect amphibian behaviors such as evading natural enemies, predation, and mate searching [48]. *A. davidianus*, being a poikilothermic creature, has its gonadal maturation, ejaculation, and ovulation mainly affected by WT and effective accumulated temperature [49]. Temperature influences the secretion and synthesis of pituitary gonadotropins, the sensitivity of gonads to gonadotropins, and the synthesis and secretion of sex hormones [50]. The optimal WT for gonadal development in *A. davidianus* ranges from 18 to 23 °C [51], with the appropriate WT for incubation ranging from 19 to 21 °C [52]. Throughout the breeding period, the WT was recorded in the experimental range of 16.9 to 22.8 °C, closely approximating the optimal temperature range, a key factor underpinning successful oviposition. Furthermore, water quality also impacts the growth and reproduction of *A. davidianus*. Throughout this study, the DO in the stream ranged from 5.50 to 9.25 mg/L, which was compatible with *A. davidianus*’s inclination toward high DO [53]. Adequate levels of DO are essential for successfully hatching *A. davidianus*’s fertilized eggs in their natural environment and for the robust development of larvae as they respire with their gills [54]. The pH ranged from 6.58 to 7.70, a neutral water quality suitable for *A. davidianus*. In addition, the parental *A. davidianus* requires a diet full of nutrients, such as live fish, fish pieces, and chicken embryos. Nutrition provides the foundation for gonadal development [55]. We could improve the breeding success rate of *A. davidianus* by reasonably adjusting various environmental factors.

## 5. Conclusions

In this study, an ethogram of *A. davidianus* during the breeding period was constructed based on the PAE coding system. A total of 10 postures, 33 actions, 11 environmental factors, and 45 behavioral patterns were identified and defined, which laid the foundation for the study of ecological laws regarding *A. davidianus*’s behavior. In addition, this study first explored the ethogram of quantitative environmental factors by incorporating quantitative data on water quality and habitat factors that affect *A. davidianus* behavior, such as WT, pH, and DO, into a coding framework that more accurately expressed the environmental conditions and thresholds required during the breeding period and revealed suitable habitats for *A. davidianus* to reproduce. Our study provides a scientific basis for optimizing the breeding techniques of *A. davidianus* and protecting its wild populations. It provides a reference for constructing a quantitative ecological factor ethogram and PAE behavior coding system for amphibians.

## Figures and Tables

**Table 1:** Posture codes for *A. davidianus*.

Posture	Definition	Code
Lying	The limbs are bent and tightened, so the whole body is pressed against the cave’s bottom.	1
Groveling	The forelimbs are slightly upright, the torso and tail are close to the cave’s bottom, and there is a small distance between the head and the cave’s bottom; the top of the head has not emerged from the water.	2
Bracing	The forelimbs, or hind limbs, are extended, causing the head and trunk to maintain a certain distance from the cave’s bottom.	3
Head exposing	The head emerges above the water’s surface.	4
Bending	The head, torso, and tail form an arc.	5
Flipping	*A. davidianus* tips over from the side of his body.	6
Crawling	*A. davidianus* displaces its body by swaying its limbs back and forth.	7
Digging	*A. davidianus* repeatedly glides its four limbs along the ground and alternately planes the bottom surface back and forth.	8
Swimming	*A. davidianus* paddles in the water with its front and hind limbs, propelling its body forward.	9
Leaping	*A. davidianus* made a forward leap.	10

**Table 2:** Action codes for *A. davidianus*.

Body Position	Action	Code
Head	Rising head	1
	Lowering head	2
	Head swing	3
	Extending forward	4
	Torsion head	5
Mouth	Kissing	6
	Inhaling	7
	Exhaling	8
	Opening mouth	9
	Closing mouth	10
	Swallowing	11
	Biting	12
	Holding	13
Limbs	Forelegs stand	14
	Forelegs bend	15
	Hindlegs stand	16
	Hindlegs bend	17
	Swing back-forth	18
	Swing left-right	19
	Unilateral straight brace	20
Trunk	Bending	21
	Hunch-up	22
	Tilting	23
	Stretching	24
	Trembling	25
	Turning	26
	Twisting	27
	Wobbling	28
Tall	Contorting	29
	Swing	30
	Upwarping	31
	Extending	32
	Leaning	33

**Table 3:** Environmental codes for *A. davidianus*.

Environment	Abiotic	Biotic	Code	Attribute
Cave	√		1	Area (0.8~1.5) m^2^ Height (0.30~0.38) m
Cave mouth	√		2	Width (0.25~0.35) m High (0.30~0.35) m
Stream	√		3	Length (20~22) m Width (0.9~1.5) m
Bottom material	√		4	Sand, pebbles
Waterbody	√		5	Water depth (0.3~0.4) m WT (16.9~22.8) °C DO (5.50~9.25) mg/L pH 6.58~7.70
Island	√		6	Substrate: sand, pebbles Vegetation covers 30%
Creek bank	√		7	The slope of both sides 60~90°
Bait	√	√	8	Live fish/fish pieces/chicken embryos
Adult male		√	9	7–11 years old Weight (6.9~12.6) kg Body length (1.03~1.28) m
Adult female		√	10	
Eggs		√	11	Bead-like

Note: √ indicates the selection of “Abiotic” or “Biotic” environments.

## Data Availability

The data presented in this study are available within this article.

## Supplementary Materials

The following supporting information can be downloaded at: https://www.mdpi.com/article/10.3390/ani13233632/s1, Video S1: The process in which the male touches the junction of the tail and genitals of the female when ovulating. Video S2: The process of the male shaking the egg pile.

## Appendix A

**Table A1:** The ethogram of *A. davidianus* during the reproductive period is based on the PAE coding system.

Behaviors	Males	Females	Time (Mth)	Number	PAE Code		
					P	A	E
**Sand-pushing behavior**							
Head pushing	+		7–8	1	7	2, 3, 4, 15, 17, 18	1, 4, 5, 9
Limbs pushing	+++		7–8	2	7, 8	15, 17, 18, 21, 27	1, 4, 5, 9
Trunk pushing	+		7–8	3	3, 7	14, 15, 16, 18, 21, 23, 27	1, 4, 5, 9
Tail pushing	+		7–8	4	7, 8	15, 17, 18, 21, 27, 29, 30	1, 4, 5, 9
**Showering behavior**							
Head showering	++	+	7–8	5	2	3, 4, 15, 17	3, 5, 9
Trunk showering	++	+	7–8	6	2, 7	15, 17, 18, 21, 24	3, 5, 9
Tail showering	++	+	7–8	7	2	15, 17, 29, 32, 33	3, 5, 9
Bend showering	+	+	7–8	8	5, 7	15, 17, 18, 21	3, 5, 9
Rising-head showering	+	+	7–8	9	3, 4	1, 2, 3, 4, 7, 8, 9, 10, 14, 17	3, 5, 9
**Courtship behavior**							
Side-by-side	+++	+++	7–8	10	2, 3	4, 14, 15, 17, 24	1, 2, 3, 5, 9, 10
Knocking bellies	++		7–8	11	7	2, 3, 4, 15, 17, 18, 19	1, 5, 9, 10
Riding	+		7–8	12	3, 7	1, 4, 14, 16, 18	1, 5, 9, 10
Following	+		7–8	13	7	3, 4, 5, 15, 17, 18	1, 5, 9, 10
Rolling over	+		7–8	14	3, 6	14, 16, 18, 19, 20, 21, 22, 23, 26	1, 5, 9, 10
Kissing	+	+	7–8	15	2, 7	3, 4, 6, 15, 17, 18	1, 2, 5, 9, 10
Inviting	++		7–8	16	7	3, 4, 15, 17, 18	1, 2, 3, 5, 9, 10
Cohabiting	+++	+++	7–8	17	1, 2	15, 17	1, 5, 9, 10
Cloacal sniffing	++		7–8	18	7	2, 3, 4, 6, 15, 17, 18	1, 5, 9, 10
**Oviposition behavior**							
Riding	++	+	8	19	3, 7	1, 4, 14, 16, 18	1, 5, 9, 10
Kissing	+	+	8	20	2, 7	3, 4, 6, 15, 17, 18	1, 5, 9, 10
Knocking bellies	++		8	21	7	2, 3, 4, 15, 17, 18, 19	1, 5, 9, 10
Intertwining	++	++	8	22	5, 7	1, 2, 3, 4, 5, 15, 17, 18, 19, 21, 27, 29	1, 5, 9, 10
Ovulating		+	8	23	2, 3, 7	15, 17, 18, 21, 24, 25	1, 5, 9, 10
Ejaculating	+		8	24	3, 7	15, 17, 18, 21, 24	1, 5, 9, 10
Fondling	+		8	25	5, 7	1, 2, 3, 4, 5, 6, 15, 17, 18, 29, 31, 33	1, 5, 9, 10
Entwining eggs		++	8	26	5, 7	1, 2, 3, 4, 5, 15, 17, 18, 21	1, 5, 9, 10
Detaining	+		8	27	3, 7	1, 2, 3, 4, 6, 9, 10, 13, 14, 15, 16, 17, 18	1, 2, 5, 9, 10
Cloacal sniffing	+		8	28	7	2, 3, 4, 6, 15, 17, 18	1, 5, 9, 10
Vigilance	++		8	29	3	4, 14, 17, 24	1, 2, 5, 9, 10
**Parental care**							
Tail fanning	+++		8–10	30	3	4, 14, 16, 24, 29, 30	1, 5, 9, 10, 11
Agitating	++		8–10	31	3, 7	1, 2, 3, 4, 14, 15, 16, 17, 18	1, 5, 9, 10, 11
Shaking	+		8–10	32	3, 7	1, 2, 3, 4, 14, 16, 18, 21, 23, 24, 28	1, 5, 9, 10, 11
Eggs eating	+		8–10	33	3	1, 2, 3, 4, 9, 10, 11, 12, 13, 14, 17	1, 5, 9, 10, 11
**Locomotive behavior**							
Chasing	+	+	7–10	34	7, 9	4, 15, 17, 18	1, 2, 3, 5, 9, 10
Following	+	+	7–10	35	7	3, 4, 5, 15, 17, 18	1, 2, 3, 5, 9, 10
Retreating	+	+	7–10	36	7	2, 3, 15, 17, 18, 19	1, 2, 3, 5, 9, 10
Swimming	+	+	7–10	37	9	4, 15, 17, 18, 30	1, 2, 3, 5, 9, 10
**Ingestive behavior**							
Capturing	+	+	7–10	38	10	1, 4, 9, 10, 12, 14, 17	1, 2, 3, 5, 8, 9, 10
Swallowing	+	+	7–10	39	2, 3	11, 12, 13, 14, 15, 17	1, 2, 3, 5, 8, 9, 10
**Resting behavior**							
Resting with bending	++	++	7–10	40	1, 2, 5	2, 15, 17, 21	1, 5, 9, 10
Resting with head-exposed	+	+	7–10	41	3, 4	1, 4, 14, 15, 17	1, 5, 9, 10
Resting with lying	+++	+++	7–10	42	1, 2	2, 4, 15, 17, 24	1, 5, 9, 10
**Miscellaneous behavior**							
Vigilance	++	++	7–10	43	3	4, 14, 17, 24	1, 2, 5, 9, 10
Rolling over	+	+	7–10	44	3, 6	14, 16, 18, 19, 20, 21, 22, 23, 26	1, 5, 9, 10
Breathing	++	++	7–10	45	3, 4	1, 2, 4, 7, 8, 9, 10, 14, 17, 24	1, 2, 3, 5, 9, 10

Note: + indicates the behavior is likely to occur, and more + indicates a higher frequency of the behavior.

## References

[1] Lorenz K.Z. (1973). The fashionable fallacy of dispensing with description. Sci. Nat..

[2] Tinbergen N. (1963). On aims and methods of ethology. Z. Tierpsychol..

[3] Lehner P.N. (1996). Handbook of Ethological Methods.

[4] Jiang Z.G. (2000). Behavior coding and ethogram of the Pere David’s deer. Acta Theriol. Sin..

[5] McDonnell S.M., Poulin A. (2002). Equid play ethogram. Appl. Anim. Behav. Sci..

[6] Settle R.A., Ettling J.A., Wanner M.D., Schuette C.D., Briggler J.T., Mathis A. (2018). Quantitative Behavioral Analysis of First Successful Captive Breeding of Endangered Ozark Hellbenders. Front. Ecol. Evol..

[7] Takahashi M.K., Okada S., Fukuda Y. (2017). From embryos to larvae: Seven-month-long paternal care by male Japanese giant salamander. J. Zool..

[8] Gong Y., Shu G., Huang F., He L., Li C., Xie F. (2018). Courtship behaviour and male sexual competition of the Taliang crocodile newt, *Liangshantriton taliangensis*. Amphibia-Reptilia.

[9] Hernandez A., Pomchote P., Jamin A. (2022). First reproduction of Panha’s crocodile newt *Tylototriton panhai* in captivity, with a description of the courtship behaviour, eggs and larval development. Herpetol. Bull..

[10] Qiao Z.L., Zhang H.H., Ma J.Z., Wei Q.G. (2015). PAE coding ethogram in breeding of semi-free-ranging Amur tiger (*Panthera tigris altaica*). Chin. J. Ecol..

[11] Liu H., Lü X., Wang X., Kou W., Miu G., Yuan H. (2021). Behavioral ethogram and posture-act-environment coding system of *Capricornis sumatraensis*. Biodivers. Sci..

[12] Wang C.B., Huang Y., Dong X., Li J.G., Zhou C.Q. (2017). Ethogram and PAE coding system of *Rostratula benghalensis* in breeding period. Sichuan J. Zool..

[13] Liu P.Z., Chen J.Z., Fan R., He Y., Zhang Y., Lu K., Zhang X.M., Zeng Q., Lei G.C. (2023). Ethogram and PAE (Posture-Act-Environment) coding system of Scaly-Sided Merganser during winter. Chin. J. Wildl..

[14] Qi Y., Li S., Suo L., Li H., Wang Y. (2011). An Ethogram of the Toad-headed Lizard *Phrynocephalus vlangalii* during the Breeding Season. Asian Herpetol. Res..

[15] Chen R., Wei Y.L., Wu L., Zheng B.Y., Li J.H. (2015). PAE coding system-based ethogram of Yangtze finless porpoise (*Neophocaena phocaenoides asiaeorientalis*) in a semi-natural environment. Acta Theriol. Sin..

[16] Zhu T.B., Yan W.B., Yang D.G. (2018). PAE coding system-based ethogram of *Schicothorax wangchiachit*. J. Fish. Sci. China.

[17] Blanchard E. (1871). On a new gigantic salamander (*Sieboldia Davidiana*, Blanch.) from Western China. Ann. Maga. Nat. Hist..

[18] Bòulenger E.G. (1924). On a new giant salamander, living in the society’s gardens. Proc. Zool. Soc. Lond..

[19] Chai J., Lu C.-Q., Yi M.-R., Dai N.-H., Weng X.-D., Di M.-X., Peng Y., Tang Y., Shan Q.-H., Wang K. (2022). Discovery of a wild, genetically pure Chinese giant salamander creates new conservation opportunities. Zool. Res..

[20] Gong Y.A., Xu J.C., Song H., Yi H.R., Li J.Q., Jiang Y.Q., Yang D.C., Yu J., Zhang Y., Li W.J. (2023). A New Species of the Giant Salamander of the Genus Andrias from Qimeng, Anhui, China (Amphibia: *Cryptorchiidae*). Chin. J. Zool..

[21] Zhang K.J., Wang X.M., Wu W., Wang Z.H., Huang S. (2002). Advances in conservation biology of Chinese giant salamander. Biodivers. Sci..

[22] Chen S., Cunningham A.A., Wei G., Yang J., Liang Z.Q., Wang J., Wu M.Y., Yan F., Xiao H.B., Harrison X.A. (2018). Determining threatened species distributions in the face of limited data: Spatial conservation prioritization for the Chinese giant salamander (*Andrias davidianus*). Ecol. Evol..

[23] Wang X.M., Zhang K.J., Wang Z.H., Ding Y.Z., Wu W., Huang S. (2004). The decline of the Chinese giant salamander *Andrias davidianus* and implications for its conservation. Oryx.

[24] Liang Z.Q., Zhang S.H., Wang C.R., Wei Q.W., Wu Y.A. (2013). Present situation of natural resources and protection recommendations of *Andrias davidianus*. Freshw. Fish..

[25] Liang G., Geng B., Zhao E. (2004). *Andrias* *davidianus*. The IUCN Red List of Threatened Species.

[26] National Forestry and Grassland Administration of China Official Release of the Updated List of Wild Animals under Special State Protection in China. http://www.forestry.gov.cn/main/586/20210208/095403793167571.html.

[27] Zhang H.X., Wang K.F., Quan Q.Z., Fan W.D., Fang S.M. (2006). Productive ecology and behavior of Chinese giant salamander (*Andrias davidianus*). J. Shaanxi Norm. Univ. Nat. Sci. Ed..

[28] Liang G., Wu F. (2010). The activity rhythm and reproductive behaviors of *Andrias davidianus*. Chin. J. Zool..

[29] Luo Q.H., Tong F., Song Y.J., Wang H., Du M.L., Ji H.B. (2018). Observation of the breeding behavior of the Chinese giant salamander (*Andrias davidianus*) using a digital monitoring system. Animals.

[30] Luo Q.H., Fu L., Jiang W.S., Zhou L.Q., Cao W., Tian H., Chen R.G. (2021). Effects of water quality on the reproductive behavior and capacity of *Andrias davidianus* under tourism disturbance. Chin. J. Appl. Ecol..

[31] Xu W.G. (2013). Showering and Courtship Behavior and PAE Coding of *Andrias davidianus* during Early Reproductive Period. Master’s Thesis.

[32] Luo M.L., Hu L.J., Shi H.T., Lin L. (2023). Ethogram and PAE Coding System of Captive Beale’s Eyed Turtle (*Sacalia bealei*). Sichuan J. Zool..

[33] Flannigan G., Stookey J. (2002). Day-time time budgets of pregnant mares housed in tie stalls: A comparison of draft versus light mares. Appl. Anim. Behav. Sci..

[34] Terry J., Taguchi Y., Dixon J., Kuwabara K., Takahashi M.K. (2019). Preoviposition paternal care in a fully aquatic giant salamander: Nest cleaning by a den master. J. Zool..

[35] Xu W.G., Wang Q.Z., Liang G. (2013). The washing behavior and its significance for male adult *Andrias davidianus* in the pre-reproductive period. Chin. J. Zool..

[36] Chen Y.X., Wang W.J., Jiang H., Yang A.S., Bai H.Q., Wu Z.C. (2007). Comparative experiments on the growth rate of male and female *Andrias davidianus*. J. Hydroecol..

[37] Okada S., Fukuda Y., Takahashi M.K. (2015). Paternal care behaviors of Japanese giant salamander *Andrias japonicus* in natural populations. J. Ethol..

[38] Unger S., Jachowski C.M.B., Diaz L., Williams L.A. (2020). Shelter Guarding Behavior of the Eastern Hellbender (*Cryptobranchus alleganiensis alleganiensis*) in North Carolina Streams. Southeast. Nat..

[39] Cui D.Y., Nui K.F., Luen T.C., Mouyu Y., Zhang Y.Y., Zhang J.G., Yang Y.Q. (2014). Behavior coding and ethogram of Guizhou snub-nosed monkey (*Rhinopithecus brelichi*). Sichuan J. Zool..

[40] Zhang J.D., Li Y., Hunag J.Y., Bai W.K., Zhou S.Q., Li Y.H., Zhou C. (2018). Behavior coding and ethogram of the sambar (*Rusa unicolor*) in field environment. Acta Theriol. Sin..

[41] Gao K.Q., Shubin N.H. (2003). Earliest known crown-group salamanders. Nature.

[42] Li W.L., Luo L., Li H., Wang S., Li Z.F., Zhai X.L., Xue Y. (2018). Advance on the artificial breeding technologies of Chinese giant salamander (*Andrias davidianus*). Chin. Fish. Qual. Stand..

[43] Luo Q.H. (2009). Habitat characteristics of *Andrias davidianus* in Zhangjiajie of China. Chin. J. Appl. Ecol..

[44] Wang W.L., Jang F.J., Wang B.L. (2000). Investigation on natural breeding habits of Chinese giant salamander. J. Hydroecol..

[45] Ge Y.R., Zheng H.X. (1994). Natural breeding cycle of the giant salamander (*Andrias davidianus*). J. Henan Norm. Univ. Nat. Sci. Ed..

[46] Kobara J., Ashikaga K. (1980). The Study about Protection of the Japanese Giant Salamander in Hiroshima Prefecture. J. Jpn. Assoc. Zoos Aquar..

[47] Browne R.K., Li H., Wang Z., Okada S., Hime P., McMillan A., Wu M., Diaz R., McGinnity D., Briggler J.T. (2014). The giant salamanders (Cryptobranchidae): Part B. Biogeography, ecology and reproduction. Amphib. Reptile Conserv..

[48] Ruiz-Aravena M., Gonzalez-Mendez A., Estay S.A., Gaitán-Espitia J.D., Barria-Oyarzo I., Bartheld J.L., Bacigalupe L.D. (2014). Impact of global warming at the range margins: Phenotypic plasticity and behavioral thermoregulation will buffer an endemic amphibian. Ecol. Evol..

[49] Zhang H.X., Wang K.F., Quan Q.Z., Fang S.M., Jing Y.L. (2003). The research on ecological breeding engineering technology of the Chinese giant salamander in Qinling Mountain area. Freshw. Fish.

[50] Mou H.M., Li Y., Yao J.J., Ma S. (2011). A review: Current research on biology of Chinese giant salamander. Fish. Sci..

[51] Jin Z.C. (2004). New technology of mass reproduction of *Andrias davidianus*. Hebei Fish..

[52] Li J.M. (2003). A probe into the spawning season of *Andrias davidianus*. Acta Hydrobiol. Sin..

[53] Luo Q.H., Liu Q.B., Liu Y., Luo H., Tang C.C. (2007). Preliminary study on ecological conditions in breeding den of Chinese giant salamander. Chin. J. Zool..

[54] Zhang H.X., Shen J.Z., Zhao H., Wang Q.J., Kouba A., Willard S.T. (2012). Water temperature of ecological-imitated breeding of parent *Andrias davidianus* in Qinba Mountains. J. Hydroecol..

[55] Liu J.Y., Xiao H.B., Lin X.Z., Yang Y.Q. (1993). Preliminary report on improving the oxytocin rate of *Andrias davidianus* under artificial ecological conditions. Freshw. Fish..

